# Associations between neurodivergence, learning disabilities and metabolic syndrome risk: evidence from the Health Survey for England

**DOI:** 10.1093/pubmed/fdaf027

**Published:** 2025-03-08

**Authors:** Nicola Shelton, Sofia Abdinor, Annemarie Lodder

**Affiliations:** Research Department of Epidemiology and Public Health, UCL, London WC1E 6BT, UK; UCL, London WC1E 6BT, UK; Neonatal Medicine Research Group, Imperial College London, London SW7 2AZ, UK

**Keywords:** disabilities, hypertension, obesity

## Abstract

**Background:**

A higher prevalence of metabolic syndrome conditions amongst individuals with learning disabilities is well established; however, there is mixed evidence for neurodivergent adults.

**Methods:**

Health Survey for England data 2016–19 were analysed. *N* = 32 390, 728 with neurodivergent conditions and 1419 with learning disabilities. Diabetes was self-reported and physician-diagnosed. Hypertension was assessed through blood pressure readings or reported medication use. Obesity and overweight were measured objectively. Covariates included age, sex and ethnicity and physical activity (2017–18 subsample).

**Results:**

An association between obesity and neurodivergence was found [relative risk ratio (RRR) = 1.64 95% CI = 1.54–1.76, *P* < 0.001]. The association with overweight was weaker. An association was found for diabetes (OR = 4.18, 95% CI = 3.82–4.57, *P* < 0.001) and hypertension (OR = 1.51, 95% CI = 1.40–1.64, *P* = < 0.001).

An association was found between obesity and learning disabilities (RRR = 1.70, 95% CI = 1.42–2.04, *P* = < 0.001) but not overweight. An association was found for diabetes (OR = 2.39, 95% CI = 2.01–2.85, *P* < 0.001) and hypertension (OR = 1.24, 95% CI = 1.02–1.50, *P* < 0.001).

**Conclusion:**

To mitigate metabolic syndrome risk, both adapting annual health checks available to adults with learning disabilities and adapting National Health Service weight management plans, to include neurodivergent adults is recommended.

## Introduction

Neurodivergence encompasses a variety of neurodevelopmental conditions impacting cognitive functioning, including autism spectrum condition (ASC), attention-deficit hyperactivity disorder (ADHD), dyspraxia and dyslexia. Neurotypical refers to individuals with normative neurological development and functioning.^1^ Differences between neurotypical and neurodivergent brain functions possibly affect the ability to communicate, learn and interact with others in a neurotypical manner. Although this is similar to that in individuals with learning (aka intellectual) disabilities such as Down’s syndrome, learning disabilities refer to conditions that significantly affect intellectual functioning and impair social and adaptive behaviour, whereas conditions like dyslexia or dyspraxia (learning difficulties) affect learning processes rather than general intellectual abilities.^2^

Metabolic syndrome refers to a group of health disorders, including insulin resistance, abdominal obesity and hypertension.^3^ Metabolic syndrome is a significant public health issue in the UK, with ~1 in 3 adults having hypertension and 5.6 million being diabetic.^4^^,^^5^ Almost 33% of adults had overweight, and 28% had obesity in 2019.^6^ Obesity places individuals at risk of developing diabetes and high blood pressure, which have significant repercussions.^7^ Research acknowledges that many neurodivergent individuals have co-occurring conditions, such as developing psychiatric health conditions.^8^ However, research gaps regarding the association between neurodivergent conditions and metabolic syndrome conditions (MSCs) exist.

Furthermore, it is estimated that 15% of the population is neurodivergent in the UK.^9^ Genome-wide association studies have highlighted that deletions on chromosome 16 are associated with intellectual disabilities and some ASC, which significantly correlates with obesity.^10^^,^^11^ The learning disabilities mortality review^12^ estimated that individuals with learning disabilities and/or a clinical diagnosis of autism have a higher mortality rate median age at death over 20 years lower than the general UK population. NHS England highlighted an increase in individuals with learning disabilities who were also diagnosed with ASC, from 21.4% in 2017–18 to 30.7% in 2021–22.^13^

A systematic review of 42 studies with 48 161 individuals with ADHD and 679 975 comparison subjects found a higher pooled prevalence of obesity among adults and children with ADHD (70% and 40%) than their comparison.^14^ These findings align with a meta-analysis of 43 studies, including 703 937 participants, which found an association between adults with ADHD and overweight and obesity of 1.3 times higher odds.^15^ A study in the USA showed that a raised body mass index (BMI) was associated with ADHD^16^, whereas a study in the Netherlands found no association with ADHD and metabolic syndrome or obesity.^17^ Flygare Wallen *et al*.^18^ found a high prevalence of obesity and diabetes amongst individuals with ASC and intellectual disabilities in the Swedish general population. They found that women with ASC, intellectual disabilities or Down’s syndrome had 2.8, 3.0 and 3.3 times higher odds of obesity, respectively, and men had 3.0, 3.4 and 3.0 higher odds. They also found that women with ASC, intellectual disabilities or Down’s syndrome had 1.6, 2.4 and 1.8 times higher odds of diabetes, respectively, and men had 1.7 and 2.0 times higher odds with no association in men with Down’s syndrome. The association was potentially due to poor lifestyle factors such as physical inactivity, poor diet, sedentary lifestyle and medication. Zheng *et al*.’s^19^ meta-analysis also showed a significant association between ASC and obesity. However, no significant association was identified between overweight and ASC despite it being a trajectory for obesity.

An online UK cohort study found that adults with ASD were more likely to be underweight or obese, but not overweight, compared to neurotypical adults.^20^ Their findings report a high prevalence of cardiovascular conditions amongst autistic adults due to unhealthy lifestyle behaviours, inadequate nutrition, unhealthy levels of physical activity and sleep. The study could not determine an independent risk of diabetes between males with and without autism but provided preliminary evidence that family history of diabetic conditions may contribute more to the increased risk of diabetic conditions among autistic females than lifestyle-related factors.^21^ A USA-based study identified adolescents with ASC as more likely to have obesity due to not meeting the recommended physical activity guidelines. The study identified that ASC adolescents were 60% less likely to participate in physical activity. In their analysis, overall physical activity fully attenuated the association between the neurodivergent condition and overweight. The odds of obesity were attenuated but remained statistically significant (1.77–1.33). The paper highlighted that adolescents with ASC may disengage in physical activity due to difficulties with social interactions, highlighting the need for targeted physical activity programs to prevent obesity amongst this group.^21^

In the UK, two weight loss management programmes, ‘Weightwise’ and ‘12-month challenge workbook’, are available to individuals with learning disabilities; however, neurodivergent conditions are classified as learning difficulties, not disabilities, in England.^7^^,^^22^ Obesity prevention programs are not extended to neurodivergent populations despite research identifying a high prevalence of obesity amongst them. However, the increasing prevalence of obesity in the UK population makes it difficult to determine whether neurodivergent adults are at risk of developing obesity or if the wider UK population is simply experiencing higher rates of obesity; thus, it is crucial to establish neurodiversity as a risk factor for obesity.^23^

Recognition that neurodivergent individuals are predisposed to physical health conditions exists, as highlighted in international studies. However, the relationship between these areas is not well understood, and evidence obtained can potentially improve the health outcomes of neurodivergent individuals if a strong association is established. Metabolic syndrome and neurodivergence are complex and influenced by a range of genetics and lifestyle factors. Further research is required to assess the high prevalence of these metabolic conditions amongst this population group.

## Statement of problem

### Aim

The aim is to investigate the co-occurring metabolic conditions in neurodivergent adults and adults with learning disabilities to see if neurodivergent adults or adults with learning disabilities are at greater risk in England and to analyse the associated risk factors.

### Objectives

The primary objective of this study is to examine whether neurodivergent conditions or learning disabilities are risk factors for metabolic syndrome conditions of obesity, diabetes and hypertension in a sample of the non-institutionalized general population in England. The second objective is to investigate if physical activity attenuates the risk of metabolic syndrome conditions amongst neurodivergent adults and adults with learning disabilities in a sub-sample in years in which these data were collected.

## Methods

### Setting and data

Health Survey England (HSE) is a cross-sectional survey representative of the general population living in non-institutionalized private households in England. The HSE sample is used to investigate the association between neurodivergent conditions, learning disabilities and metabolic syndrome conditions. The study utilizes data collected from 2016 to 2019, a national random sample, to examine this relationship. Data regarding the explanatory variables (neurodivergent conditions and learning disability) and outcome variables (diabetes, obesity and hypertension) are obtained from questionnaires, interviews, self-reported measures and nurse visits.

### Analytical sample

The analysis consisted of adult participants aged 16 and over, leading to a total sample size of *N* = 32 390: *N* = 8011 in 2016, *N* = 7997 in 2017, *N* = 8178 in 2018 and *N* = 8204 in 2019. The response rates for the HSE sample were 60% in 2019, 59% in 2018, 60% in 2017 and 59% in 2016.^23–26^

### Measures

Interviewers collected information regarding age, sex, ethnicity, general health, longstanding conditions (including neurodivergence and learning disabilities), self-reported doctor-diagnosed diabetes and hypertension and height and weight measurements. A sub-sample of participants provided blood pressure measurements in nurse visits. Physical activity levels in 2017 and 2018 were measured using the International Physical Activity Questionnaire (IPAQ).^27^

To conduct the quantitative research, the sample obtained from HSE was based on exclusion and inclusion criteria. Pregnant women were excluded. Participants in the ‘refused’ or ‘do not know’ categories in the explanatory and outcome variables were excluded.

Data over the 4 years were combined to increase the sample size for analysis. Demographic variables of age, sex and ethnicity were derived from questionnaires and interviews. The explanatory variables derived for neurodivergence and learning disability were self-reported by the participants. For the outcome variables: diabetes was classified as participants who self-reported a medical diagnosis. Hypertension was calculated using blood pressure measurements by a nurse or taking medication for hypertension, whilst obesity was obtained from the body mass index variable, whereby objective height and weight measurements were taken by an interviewer, excluding self-reported measures. The physical activity variable was self-reported.^23–26^

The following sections outline HSE dataset variables and their associated measurement questions.

Participants were asked: ‘Do you have any physical or mental health conditions or illnesses lasting or expected to last 12 months or more?’ They were then asked follow-up questions about the nature of the conditions including: Neurodivergent conditions (this was a single response item, with conditions not further differentiated): ‘whether conditions or illnesses affect you: socially or behaviourally’ (for example, associated with autism, attention deficit disorder or Asperger’s syndrome) and Learning Disability: ‘whether conditions or illnesses affect you: Learning, understanding or concentrating’.

#### Hypertension

Hypertension was defined as ‘whether hypertensive:140/90 (Omron readings) or prescribed drugs for BP’.

#### Diabetes

Participants were asked: ‘Do you now have, or have you ever had diabetes? Were you told by a doctor that you had diabetes?’

#### Body mass index

Height and weight were measured using a portable stadiometer and Seca 877 scales and the BMI variable derived from these using (weight in kilograms) divided by (height in metres squared). The BMI was grouped into underweight and normal; overweight; and obese according to the World Health Organization classification.^28^

#### Physical activity

The IPAQ defines activity levels based on reported moderate or vigorous physical activity (MVPA). Respondents reporting below 30 minutes MVPA per week have been defined as ‘inactive’, whereas those reporting 30 minutes or more have been defined as ‘active’. Note: this differs from the UK Chief Medical Officers’ Physical Activity Guidelines for sufficient levels of aerobic activity (at least 150 minutes/week of MVPA).^29^

### Statistical analysis

All statistical analyses were performed using Stata/MP 18.0.^30^ Data were selected, missing values and outliers were removed, and variables were recoded appropriately. Analysis was adjusted for survey design and non-response. Multinomial logistic regression was used to explore the relationship between neurodivergent conditions, overweight and obesity and learning disability, overweight and obesity, providing relative risk ratios (RRRs), 95% confidence intervals (CI) and *P*-values. Logistic regression was used to analyse the relationship between neurodivergent conditions and the prevalence of diabetes and hypertension separately. This analysis was also conducted for learning disabilities to compare their risk of metabolic conditions. Significance levels were set at a *P*-value <0.05.

### Ethics

The project did not require additional ethical approval as it utilized secondary analysis of published datasets only. Before undertaking the quantitative research and data collection, HSE was issued ethical approval from the East Midlands Nottingham 2 Research Committee in 2015.

## Results

Table 1 provides frequencies and percentages of the main characteristics of the study population over 4 years from 2016 to 2019. It presents individuals 16 and over with neurodivergent conditions and learning disabilities, including the prevalence of health conditions, hypertension, obesity and diabetes outcome variables. Age, sex and ethnicity are provided for all years and physical activity data for the years 2017 and 2018.

The sample consists of 32 390 total participants, of which 55% were female. The majority participants identified as White (87%). The small number of participants in the ‘refused’ and ‘don’t know’ categories for ethnicity (*n* = 69) were removed. The sample includes a total of 728 participants with neurodivergent conditions (2%), 1419 (4%) with learning disabilities and 17 645 (54%) without any health condition. Three respondents without health data were excluded. The data indicate a high proportion (72–73%) of individuals were physically active in the sample in the subsample from 2017 and 2018.

Table 1 shows that over one-third of (36%) of the sample had overweight and another third (29%) had obesity. The total sample size for BMI was *N* = 26 413. The prevalence of diabetes was 8%. Missing data were excluded for diabetes (*n* = 2). The prevalence of hypertension was 33% in a sample of *N* = 16 966.

Table 2 displays the association between neurodivergent conditions and learning disability with the dependent variable: overweight and obesity, adjusting for age, sex and ethnicity and, for 2017–18, additionally adjusting for physical activity. The RRR between neurodivergent conditions and overweight was 1.12 *P* < 0.001; This increased risk was also observed after additionally adjusting for physical activity RRR = 1.10 *P* = 0.047. A significant positive relationship was found between neurodivergent conditions and obesity (RRR = 1.64, *P* < 0.001). Upon adjusting for physical activity in a subsample, the association between neurodivergent condition and obesity was somewhat attenuated, with the RRR 1.42 (*P* < 0.001).

**Table 1:** Sample characteristics.

Variables	2016–19 combined	Year 2019	Year 2018	Year 2017	Year 2016
**N (%)**					
**Neurodivergence**					
No condition	17 645 (54.4%)	4485 (54.7%)	4442 (54.3%)	4304 (53.8%)	4414 (55.1%)
Other conditions	14 014 (43.3%)	3566 (43.5%)	3522 (43.1%)	3516 (44.0%)	3410 (42.6%)
Neurodivergent	728 (2.3%)	153 (1.9%)	214 (2.6%)	175 (2.2%)	186 (2.3%)
Total	32 387	8204	8178	7995	8010
**Learning disability**					
No condition	17 645 (54.5%)	4485 (54.7%)	4442 (54.3%)	4304 (53.8%)	4414 (55.1%)
Other condition	13 323 (41.1%)	3369 (41.1%)	3340 (40.8%)	3337 (41.7%)	3277 (40.9%)
Learning disability	1419 (4.4%)	350 (4.3%)	396 (4.8%)	354 (4.4%)	319 (4.0%)
Total	32 387	8204	8178	7995	8010
**Sex**					
Female	17 959 (55.4%)	4530 (55.2%)	4509 (55.1%)	4461 (55.8%)	4459 (55.7%)
Male	14 431 (44.6%)	3674 (44.8%)	3669 (44.9%)	3536 (44.2%)	3552 (44.3%)
Total	32 390	8204	8178	7997	8011
**Age**					
16–24	2853 (8.8%)	729 (8.9%)	748 (9.1%)	647 (8.1%)	729 (9.1%)
25–34	4528 (14.0%)	1083 (13.2%)	1154 (14.1%)	1068 (13.4%)	1223 (15.3%)
35–44	5245 (16.2%)	1397 (17.0%)	1294 (15.8%)	1331 (16.6%)	1223 (15.3%)
45–54	5563 (17.2%)	1416 (17.3%)	1386 (16.9%)	1354 (16.9%)	1407 (17.6%)
55–64	5372 (16.6%)	1349 (16.4%)	1342 (16.4%)	1367 (17.1%)	1314 (16.4%)
65–74	4984 (15.4%)	1242 (15.1%)	1325 (16.2%)	1239 (15.5%)	1178 (14.7%)
75+	3845 (11.9%)	988 (12.0%)	929 (11.4%)	991 (12.4%)	937 (11.7%)
Total	32 390	8204	8178	7997	8011
**Ethnicity**					
White	28 166 (87.2%)	7016 (85.8%)	7088 (86.9%)	7028 (88.2%)	7034 (88.0%)
Black	958 (3.0%)	241 (2.9%)	256 (3.1%)	218 (2.7%)	243 (3.0%)
Asian	2383 (7.4%)	713 (8.7%)	594 (7.3%)	560 (7.0%)	516 (6.5%)
Mixed/Multiple ethnic backgrounds	491 (1.5%)	133 (1.6%)	136 (1.7%)	101 (1.3%)	121 (1.5%)
Other ethnic groups	285 (0.9%)	72 (0.9%)	78 (1.0%)	59 (0.7%)	76 (1.0%)
Total	32 283	8175	8152	7966	7990
**Socioeconomic status**					
Managerial and professional	11 288	2930	2888	2810	2660
Intermediate	7587	1912	1903	1861	1911
Routine and manual	11 456	2843	2846	2857	2910
Not otherwise classified	2059	519	541	469	530
Total	32 290	8204	8178	7997	8011
**Diabetes diagnosis**					
Not diagnosed with diabetes	29 920 (92.4%)	7566 (92.2%)	7538 (92.2%)	7416 (92.8%)	7400 (92.4%)
Diagnosed with diabetes	2468 (7.6%)	638 (7.8%)	639 (7.8%)	580 (7.3%)	611 (7.6%)
Total	32 388	8204	8177	7996	8011
**Hypertension status**					
Not hypertensive	11 390 (67.1%)	2813 (67.5%)	2719 (66.9%)	2966 (67.6%)	2892 (66.5%)
Hypertensive	5576 (32.9%)	1352 (32.5%)	1347 (33.1%)	1423 (32.4%)	1454 (33.5%)
Total	16 966	4165	4066	4389	4346
**Body mass index**					
Underweight and normal	9123 (34.5%)	2244 (33.6%)	2310 (34.5%)	2198 (33.7%)	2371 (36.4%)
Overweight	9601 (36.3%)	2456 (36.8%)	2432 (36.3%)	2394 (36.7%)	2319 (35.7%)
Obese	7689 (29.1%)	1980 (29.6%)	1962 (29.3%)	1938 (29.7%)	1809 (27.8%)
Total	26 413	6680	6704	6530	6499
**Physical activity**					
Inactive	3756 (27.3%)	Not available	1908 (27.8%)	1848 (27.0%)	Not available
Active	9958 (72.6%)	Not available	4959 (72.2%)	4999 (73.0%)	Not available
Total	13 714	Not available	6867	6847	Not available

Note: Sample sizes in the regression may differ to the full sample due to missing values in the outcome or dependent variables

**Table 2:** Relative risk of overweight and obesity in adults age 16 and over, reporting neurodivergence or learning disability, England.

		Year 2016–19 (adjusted for age, sex and ethnicity)				Year 2017–18 (additionally adjusted for physical activity)			
	Sample size	RRR	*P*-value	95% CI		RRR	*P*-value	95% CI	
**Overweight**									
**Neurodivergence**	***N* = 26 389**					***N* = 11 824**			
No reported neurodivergence		1				1			
Neurodivergence		1.12	0.001	1.05	1.19	1.10	0.047	1.00	1.21
**Learning disability**	***N* = 26 391**					***N* = 11 825**			
No reported learning disability		1							
Learning disability		1.16	0.121	0.96	1.41	1.14	0.121	0.86	1.51
**Obesity**									
**Neurodivergence**	***N* = 26 389**					***N* = 11 824**			
No reported neurodivergence		1							
Neurodivergence		1.64	<0.001	1.54	1.76	1.42	<0.001	0.86	1.68
**Learning disability**	***N* = 26 391**					***N* = 11 825**			
No reported learning disability		1							
Learning disability		1.70	<0.001	1.42	2.04	1.45	0.005	1.12	1.88

There was no significant association between learning disability and overweight before and after adjusting for physical activity (*P* = >0.05). A significant association was found between learning disability and obesity before (RRR = 1.70, *P* < 0.001) and after (RRR = 1.45, *P* = 0.005) being adjusted for physical activity.

Table 3 presents the relationship between neurodivergence and learning disabilities with diabetes. Results indicate a significant relationship between diabetes and neurodivergent conditions and learning disability, respectively, with an OR of 4.18 (*P* < 0.001) and 2.39 (*P* < 0.001). Despite the slight decrease in OR after additionally adjusting for physical activity, there was still a significant association between having diabetes in adults with neurodivergent condition (OR = 3.57, *P* < 0.001) or a learning disability (OR = 1.50, *P* = 0.016).

**Table 3:** Odds ratio of diabetes in adults age 16 and over, reporting neurodivergence or learning disability, England.

Diabetes		Year 2016–19 (adjusted for age, sex and ethnicity)				Year 2017–18 (additionally, adjusted for physical activity)			
	Sample size	OR	*P*-value	95% CI		OR	*P*-value	95% CI	
**Neurodivergence**	***N* = 32 278**					***N *= 13 692**			
No reported neurodivergence		1				1			
Neurodivergence		4.18	<0.001	3.82	4.57	3.57	<0.001	3.06	4.17
**Learning disability**	***N* = 32 281**					***N* = 13 694**			
No reported learning disability		1				1			
Learning disability		2.39	<0.001	2.01	2.85	1.50	0.016	1.08	2.08

Table 4 also reveals a strong association between neurodivergent conditions and the prevalence of hypertension (OR = 1.51, *P* = < 0.001). After additionally adjusting the model for physical activity, the association was similar (OR = 1.45, *P* < 0.001). Table 4 shows that individuals with learning disabilities had a significantly higher odds of hypertension before (OR = 1.24, *P* < 0.001) but not after adjusting for physical activity (*P* > 0.05).

**Table 4:** Odds ratio of hypertension in adults age 16 and over, reporting neurodivergence or learning disability, England.

Hypertension		Year 2016–19 (adjusted for age, sex and ethnicity)				Year 2017–18 (additionally, adjusted for physical activity)			
	Sample size	OR	*P*-value	95% CI		OR	*P*-value	95% CI	
**Neurodivergence**	***N* = 16 953**					***N* = 7569**			
No reported neurodivergence		1				1			
Neurodivergence		1.51	<0.001	1.40	1.64	1.45	<0.001	1.29	1.62
**Learning disability**	***N* = 16 954**					***N* = 7569**			
No reported learning disability		1				1			
Learning disability		1.24	<0.001	1.02	1.50	1.29	0.087	0.96	1.73

## Discussion

### Main finding of this study

Neurodivergent adults and adults with learning disabilities in England have a higher risk of metabolic syndrome conditions.

### What is already known on this topic

Prior research highlighted a significant association between neurodivergent adults and adults with learning disabilities having an elevated risk of MSCs. Lower levels of physical activity (or low physical activity and other lifestyle factors) increased the risk of having overweight and obese in autistic adults.

### What this study adds

There were similar higher risks of having obesity in neurodivergent adults and adults with learning disabilities but not overweight. Neurodivergent adults had somewhat higher risk of hypertension compared with adults with learning disabilities. Neurodivergent adults had considerably higher risk of diabetes compared with adults with learning disabilities. The attenuation of risk by physical activity varied by condition and differed between neurodivergent adults and adults with learning disabilities.

The results of this study highlight neurodivergence individuals to be at similar or greater risk as individuals with learning disabilities in having metabolic syndrome conditions. These findings underline the importance of interventions tailored to the neurodivergent population in England to reduce their risks of having obesity, diabetes and hypertension.

### Limitations of this study

As with many studies assessing the relationship between neurodivergent conditions and the prevalence of metabolic syndrome, data on lifestyle behaviour variables, such as nutrition, were not available. These behavioural factors could be confounding variables, influencing the relationship between the explanatory and outcome variables. The survey has not included information on eating habits in adults since 2008 (other than fruit and vegetable consumption in certain years). Therefore, this study did not include information about other nutrients, daily calorie intake or portion sizes, which influence obesity, diabetes and hypertension.

Despite the HSE sample being representative of non-institutionalized households, people without stable housing and institutionalized individuals, including prisons or long-term care settings, are not represented. This is notable as individuals with disabilities are more likely to be in institutional care^31^ and neurodivergent populations are more likely to be in prisons^32^ or mental health hospitals.^33^ These exclusions could limit the generalizability of the results, particularly for populations who may face unique risk factors for metabolic syndrome conditions such as limited autonomy over their diet and physical activity levels. The true problem of metabolic syndrome conditions in neurodivergent populations may be underestimated.

Future research should address these gaps by including participants who represent institutionalized individuals and those without stable housing. This could involve collecting data from prisons, residential care facilities and homeless shelters to ensure that the experiences of these groups are captured. Additionally, targeted studies could explore how environmental factors, such as limited access to nutritious food or healthcare, increase metabolic syndrome conditions risk in these underrepresented populations. Expanding the scope of research in this way would enhance our understanding of the full range of risks faced by neurodivergent people and those living with learning disabilities.

Only 2 years of physical activity data were available using the same methodology, which may introduce bias in the form of over- or underestimation of the relationship between physical activity and development of health conditions in adults with neurodivergent conditions. Self-reported questionnaires, HSE 2017 and 2018, present risk of recall bias and altering responses to conform to social norms, impacting results.

Moreover, the questionnaire did not ask which neurodivergent conditions individuals had, instead grouping them together as a single item response. This makes it impossible to determine from this dataset whether adults with specific neurodivergent conditions are more at risk of metabolic syndrome than others.

## Conclusion

In conclusion, this study has sought to fill a gap in England’s research surrounding the association between neurodivergent conditions and metabolic syndrome conditions. This research has identified neurodivergent individuals, like those with learning disabilities, are at a greater risk of diabetes and hypertension compared to healthy counterparts. This research identifies neurodivergent individuals as at greater risk of being obese after adjusting for physical activity, suggesting that additional interventions alongside physical activity, for example weight management plans should be developed and introduced to mitigate the risk. Similarly, despite existing programs, adults with learning disabilities maintain a higher risk of having obesity; therefore, current interventions for this group should be improved.

As neurodivergent adults are at a higher risk of having obesity, prevention strategies targeting physical activities should be considered with this population group in mind to reduce the prevalence of overweight. An example of a practical approach would be including them in the weight loss strategy proposed by Public Health England,^7^ which currently includes individuals with learning disabilities. However, physical activity alone may not be sufficient to mitigate the risk of obesity, and additional interventions may be required due to a significant association observed independently between neurodivergence and learning disabilities with obesity after adjusting for physical activity. It could be suggested that early interventions targeting the two population groups, specifically when they have overweight, to prevent obesity should be adopted.

NHS weight management programmes currently support people with learning disabilities. Several challenges will need to be considered to extend these programmes to neurodivergent people and to reduce barriers to participation. A qualitative study by Healy *et al*.^34^ found that some autistic traits in particular can be barriers to weight management such as social anxiety, sensory sensitivity, obsessiveness and a strong desire for routine. Additionally, neurodivergent people, like those with ASC, may feel a disconnect between how they perceive their body and how it actually looks in real life. Weight management programmes should incorporate sensory-friendly meals, be able to provide information and support online and be flexible to increase programme accessibility. This would require additional funding, staff training and programme modifications. Feasibility studies should be conducted to assess the feasibility and acceptability of such modified interventions. Uncontrolled hypertension and diabetes pose major health risks. Hypertension can now be measured easily, and affordable, portable devices are available for home use. The HSE asks about self-reported doctor-diagnosed diabetes, so those adults should be under the care of a general practitioner (GP) already, but the gap is in checks for those undiagnosed and untreated. Suitable programmes could significantly improve health outcomes in neurodivergent populations and reduce NHS costs by preventing long-term complications associated with metabolic syndrome.

The NHS recommends physical activity and maintaining a healthy weight to reduce hypertension.^24^ This research suggests that physical activity alone is not enough to mitigate the risk of having diabetes amongst individuals with neurodivergent conditions and learning disabilities and hypertension in neurodivergent adults but by taking a more comprehensive approach and additionally offering support. Some neurodivergent adults demonstrate sensory difficulties and are more selective with what they consume depending on texture, smell and food temperature.^21^ This results in diets that may not fit the standard form of healthy eating. Thus, health care professionals should provide support and encouragement to identifying healthy food options that are not triggering in order to maintain a healthy lifestyle, reducing their risk of diabetes^35^ [and obesity and hypertension].

These findings emphasize the importance of primary care providers being aware that neurodivergent adults are at higher risk of developing conditions associated with metabolic syndromes and extend weight loss management programmes targeted at individuals with learning disabilities should be adapted to be suitable for neurodivergent populations. Physicians should also support these population groups’ health literacy regarding lifestyle behaviours to reduce their risks of developing metabolic syndrome conditions. In the UK, adults age 14 and over with learning disabilities are offered an annual health check, but, in the UK, this does not currently include neurodivergent adults due to these conditions not being formally included in this classification. Consultations have been heard regarding extending annual health checks to autistic adults, and a pilot is underway.^36^ Annual health checks should be made available to neurodivergent adults to mitigate the risk of metabolic syndrome conditions.

## Data Availability

Anonymized datasets Study Numbers 8334, 8488, 8649 and 8860 were obtained from the UK Data Service through university institutions and users agreeing to licence conditions.^37^

## Notes

**Nicola Shelton,** Professor of Population Health

**Sofia Abdinor,** Graduate BSc Population Health Sciences

**Annemarie Lodder,** Research Fellow
